# Emission and Mechanical Properties of Glass and Cellulose Fiber Reinforced Bio-Polyamide Composites

**DOI:** 10.3390/polym15122603

**Published:** 2023-06-07

**Authors:** Susanne Wolff, Annette Rüppel, Hassan Ali Rida, Hans-Peter Heim

**Affiliations:** Institute of Material Engineering, Polymer Engineering, University of Kassel, 34125 Kassel, Germany; annette.rueppel@uni-kassel.de (A.R.); h.rida@uni-kassel.de (H.A.R.); heim@uni-kassel.de (H.-P.H.)

**Keywords:** fiber reinforced composites, bio-polyamide, glass fiber, cellulosic fiber, VOC analysis, emission, sensory evaluation, odor, mechanical properties

## Abstract

Climate change, access, and monopolies to raw material sources as well as politically motivated trade barriers are among the factors responsible for a shortage of raw materials. In the plastics industry, resource conservation can be achieved by substituting commercially available petrochemical-based plastics with components made from renewable raw materials. Innovation potentials are often not used due to a lack of information on the use of bio-based materials, efficient processing methods, and product technologies or because the costs for new developments are too high. In this context, the use of renewable resources such as fiber-reinforced polymeric composites based on plants has become an important criterion for the development and production of components and products in all industrial sectors. Bio-based engineering thermoplastics with cellulose fibers can be used as substitutes because of their higher strength and heat resistance, but the processing of this composite is still challenging. In this study, composites were prepared and investigated using bio-based polyamide (PA) as a polymer matrix in combination with a cellulosic fiber and, for comparison purposes, a glass fiber. A co-rotating twin-screw extruder was used to produce the composites with different fiber contents. For the mechanical properties, tensile tests and charpy impact tests were performed. Compared to glass fiber, reinforced PA 6.10 and PA 10.10, a significantly higher elongation at break with regenerated cellulose fibers, can be achieved. PA 6.10 and PA 10.10 achieve significantly higher impact strengths with the regenerated cellulose fibers than the composites with glass fibers. In the future, bio-based products will also be used in indoor applications. For characterization, the VOC emission GC-MS analysis and odor evaluation methods were used. The VOC emissions (quantitative) were at a low level but the results of the odor tests of selected samples showed values mostly above the required limit values.

## 1. Introduction

Due to their valuable properties, fiber-reinforced thermoplastics have a wide range of applications. Possible areas of application can be found in the fields of the automotive industry, aeronautic industry, and the household sector. The largest material group within the thermoplastic composites, but also in the overall market, are the short fiber reinforced plastics. For a wide range, standard polymers such as PP are used. However, where higher mechanical and thermal properties are required, engineering polymers such as polyamides (PA) are preferred [1,2,3,4].

Bio-based polymers with reinforced fibers have found their way into the market and ensured potential growth in international markets as a replacement for various materials. This alternative way of introducing fiber-reinforced composites is to provide safer and environmentally friendly products [5,6,7]. Bio-polyamides (e.g., PA 10.10), which are synthesized from up to 100% renewable raw materials, are now competitive with petroleum-based plastics from an economic point of view. Especially since the properties of bio-polyamides are very similar or even superior to those of conventional polyamides. For example, they are characterized by high mechanical and thermal stability, high dimensional stability, lower water absorption, low gas and vapor permeability, high impact strength, and lower density [5,8,9].

The use of natural fibers in composite materials is increasing due to legislation forcing automotive manufacturers to reuse and recycle materials, which is leading to an increase in the bio-based material content in automotive applications and can be a suitable alternative for petroleum-based materials [1,10]. Natural fibers can be obtained from various plants or parts of plants, e.g., roots, stems, leaves, fruits, or seeds. Many common plant fibers such as abaca, cotton, coconut, bamboo, hemp, jute, rattan, and others are known. In this context, wood, bamboo, and rattan represent the world’s three largest forestry resources for the use of renewable raw materials [10,11] Cellulose can be obtained by various manufacturing processes. The cellulose production process influences the properties of the cellulose and thus also the properties of the subsequent construction materials. For example, Han et al. showed in a recent study that the tensile strength and toughness of cellulose materials gradually improved with increasing moisture content. This proved that water molecules play an active role in the production of strong and tough cellulosic materials. This ultrastrong and tough cellulosic material was fabricated by a two-step process of delignification and water molecule-induced hydrogen bonding under compression [12]. By using the viscose process, cellulose from wood, plant fibers, and cotton is spun into yarn and then regenerated into cellulose-regenerated fibers (CRF). CRF have many advantages over technically important natural fibers such as flax, jute, hemp, and sisal which result from the technical manufacturing process (man-made fiber). To reinforce engineering polymers with natural fibers, cellulose fibers with higher thermal stability are required. The most important segment in the chemical–technical processing of cellulose is represented by products made of regenerated cellulose. Viscose fibers have excellent properties for a broad product range, from wet-strength cotton-like textile fibers to technical fibers. The regenerated cellulosic fibers have a higher ductility and a higher thermal resistance compared to natural fibers [13,14,15]. In some cases, CRF has mechanical properties that exceed natural fibers, such as strength and toughness. Furthermore, CRF only exhibits minor variations in properties [16].

The main problem in plastic processing with natural fibers is the obligatory high processing temperature of the matrix material. Natural fibers generally consist of a mixture of cellulose, hemicelluloses, lignin, waxes, and other natural accompanying substances. Depending on the plant of origin and from which part of the plant the material was obtained, the percentages of cellulose, hemicellulose, and lignin vary and influence the properties of the fibers and composites [8,10,11,17]. Natural fibers tend to release volatile organic compounds (VOCs) even at low temperature stress. These are generally undesirable and limit the application areas of natural fibers as well as composite materials. As cellulosic regenerated fibers consist only of cellulose and do not contain any low molecular or volatile compounds, CRF can be used at higher processing temperatures. Besides fiber degradation, degradation of the matrix material can also lead to emissions [8,18,19,20,21]. The limit value for the chemical-thermal degradation of natural fibers is around 200 °C, depending on the main constituent, lignin, and hemicellulose. This is contrary to cellulose, which degrades above 300 °C. Feldmann found that significant temperature-induced bulk degradation of a regenerated cellulose fiber was observed between 240–260 °C. Particularly affected by this is the compounding of polyamide, which requires significantly higher temperatures (melting temperature 250 °C) compared to polyolefin matrix polymers (e.g., PP, PE, or PLA). Processing without loss of mechanical properties is only possible if narrow boundary conditions are observed in the process control [16,22,23].

Compared to glass fibers (GF), CRF has a low density (1.5 versus 2.5 g/cm^3^). This results in a high lightweight potential for CRF-reinforced composites, which can also be partially or, with the selection of suitable matrices, even completely bio-based [24,25]. CRF-reinforced plastics also exhibit better acoustic properties (damping) and a reduced tendency to splinter in the event of a failure than glass fiber-reinforced counterparts. Furthermore, cellulose-regenerated fibers experience significantly less length reduction during processing, which means that the composites made from them have a high recycling potential [26]. This means that after several processing cycles, the mechanical properties of cellulose fiber-reinforced composites are maintained at a high level, whereas this is not the case for glass fiber-reinforced plastics after a single processing cycle. Cellulose fibers are characterized by a significantly narrower strength distribution, which increases the reinforcement efficiency compared to glass fibers and, on the other hand, reduces the property variations of the composite materials [27].

Standard polymers such as polypropylene (PP) and polylactic acid (PLA) are very well suited for reinforcement with natural and cellulosic fibers. There are numerous studies that show the mechanical, thermal, and acoustic properties of fiber-reinforced PP and PLA with natural and cellulosic fiber [13,27,28,29,30,31,32]. When higher demands are made on the mechanical and thermal technical polymers are used. Many studies of cellulosic fiber-reinforced composites with polyamides (PA), polybutylene terephthalate (PBT), and polytrimethylene terephthalate (PTT) were investigated and accomplished for this task [32,33,34,35,36].

The process conditions for extrusion and injection molding of polyamide composites with regenerated cellulose fibers, glass fibers, and abaca fibers have been investigated in various studies with respect to mechanical and thermal properties. Feldmann et al. examined the influence of the processing parameters on the mechanical properties of polyamides with regenerated cellulose fibers using a twin-screw extruder. Significant increases in impact and tensile properties were achieved on regenerated cellulose fiber (CRF) with bio-based polyamide. Moreover, CRFs show a higher thermal resistance than abaca fiber. Furthermore, the study has shown that the temperatures and screw configurations of the twin-screw extruder only result in different fiber length distributions [14,23,37]. Klason et al. investigated composites consisting of thermoplastics (HDPE, PP, PS, SB, PA 6, and PA 12) and cellulose-based fillers (wood flour, cellulose flour, and cellulose fiber). They found an increase in modulus with increasing filler content, while the yield and breaking stress remained relatively unaffected. For PA 12, a moderate increase in the strength level was noted. The extension at rupture and the impact strength fell sharply when the filler content was increased. The compounding method had no influence on the modulus [38].

In addition to mechanical and thermal properties, VOC emissions are equally important, especially when products are used in interiors. Polymer materials, additives, reinforcing materials, and others exhibit a thermally conditioned emission behavior. These thermal behavior changes are responsible for the emission of volatile organic compounds (VOCs), which have been getting attention in recent years. The concentration of VOCs affects and influences the quality of air and odor in vehicles. The emission of semi-volatile organic compounds (SVOCs) leads to what is known as “Fogging”, a blur on the inside of the windshield that disables the driver of his visibility [39,40,41].

In the literature, there is very limited information about the odor and emission properties of cellulose fiber-reinforced bio-polyamide composites. However, other material combinations have already been studied and the results have been published. Kim et al. (2006) evaluated the VOC emissions from building finishing materials (wood-based composites). The research indicated that emissions of VOCs from the composites could adversely affect indoor air quality [42]. Lee et al. (2008) investigated bio-composites, formaldehyde, and TVOC emission. Based on the result, the TVOC emission level is very low in all of the bio-composite samples except the formaldehyde and TVOC emission level of the bio-composites with the attached veneer [43]. Khoshnava et al. evaluated the VOC emissions of conventional building materials and green building materials. The obtained result exposed that the total indoor impacts of TVOCs on human health incredibly decline with the substitution of bio-composites for petroleum-based composites. The TVOC emission rate from bio-composites is acceptable according to a different standard, but this is not true for petroleum-based composites [44].

Kriek et al. examine different PA 6 and PA 6.6 grades. The main emissions could be identified depending on extrusion conditions. Among others, caprolactam is also one of the main emissions from polyamide 6 and its copolymers. The data allow manufacturers to be used as reference points to estimate emissions from similar polyamide resins processed on similar equipment [45]. Courgneau et al. studies show that different stages of processing (drying cycles, compounding, injection molding) affect the degradation of PLA and bio-composites properties. The changes in molecular weight and global odor emission indicated that compounding had a strong impact on PLA degradation and odor emission, while injection molding had no significant impact [46]. Rüppel et al. examine the impact of accelerated aging on the VOC emission of glass fiber reinforced polypropylene. An increase in emission after processing was observed. For polypropylene composites reinforced with glass fibers, hydrocarbon compounds (HC) with a chain length of C6-C31 have been detected [47].

In this study, short glass and cellulosic fiber-reinforced thermoplastics (bio-polyamide) were produced on a twin-screw extruder. The materials used were selected against the background of producing a composite from 100% renewable raw materials. In order to be able to use the materials in interior areas, the emissions emitted by these products should not contribute to the deterioration of the indoor air. First, glass fiber and cellulose fiber reinforced composites were investigated with regard to their mechanical properties. Since it is known from the literature that a composite with 20 wt.% cellulose fibers and 30 wt.% glass fibers have approximately the same volume fraction of fibers, this pairing in particular was investigated. The study focused on characterizing the volatile organic compounds emissions VOC from cellulose fiber-reinforced bio-polyamide using gas chromatography-mass spectrometry (GC-MS). In order to be able to determine the emitting substances, the test method had to be developed, since the standardized method did not lead to the goal. Conditioning in an emission chamber led to the goal. In addition to the development of the test method, a qualitative and quantitative analysis of the detected substances and the emission values was carried out. In addition, odor investigations were carried out for selected composites. As a result, findings regarding VOC emissions could be obtained by comparing the different composites. This supports the decision as to whether cellulose-regenerated fiber-reinforced bio polyamides can replace the glass fiber-reinforced variants for indoor use.

## 2. Materials and Methods

### 2.1. Origin and Characteristics of Materials

For the manufacturing of test specimens, a bio-polyamide (PA 10.10 and PA 6.10) with glass fibers (GF) and regenerated cellulose fibers (CRF) compounds were manufactured.

#### 2.1.1. Polymer Matrix

The matrix materials used in this study are a polyamide PA 10.10 (Vestamid Terra DS 16) and a polyamide PA 6.10 (Vestamid Terra HS 16) provided by Evonik Industries AG (Essen, Germany). According to the manufacturer, Vestamid Terra is a polyamide renewable raw material. The monomers are obtained partly or entirely from castor oil.

Vestamid Terra DS 16 can be used for the production of injection-molded or extruded products or can be used as a component of other industrial products. It provides high mechanical strength, good UV and chemical resistance, and can be used at high temperatures. Vestamid Terra DS fills a position similar to that of the long-chain high-performance polyamides PA 12 and PA 12.12 and the standard polyamides PA 6 and PA 6.6, which have a shorter chain length. Selected properties of Vestamid Terra DS 16 and HS 16 are shown in Table 1.

#### 2.1.2. Glass Fiber

The glass fiber (GF) used in this study is an e-glass fiber type FGCS 3540 provided by Schwarzwälder Textil-Werke (Schenkenzell, Germany). The fiber sizing (silane-based) content is approx. 1.1% according to DIN ISO 1887 (sizing content determination by loss on ignition (625 °C).

#### 2.1.3. Regenerated Cellulose Fiber

The regenerated cellulose fiber used in this study is the Cordenka CR-Type (CRF) provided by Cordenka GmbH & Co. KG (Obernburg, Germany). CRF is produced by the viscose process in which bio-based cellulose from different types of wood, plant fibers, and cotton is spun into yarn in a chemical process. The fiber contains up to 0.25% of a fiber preparation (avivage), which is not named more precisely by the manufacturer.

The mechanical properties of the fibers are listed in Table 2.

### 2.2. Preparation of Test Specimens

In most commercial applications of glass fiber-reinforced thermoplastic compounds, 30 wt.% glass fibers are added. Due to the varying densities of the fibers used in this investigation, a fiber volume content *φ* of 20 wt.% CRF is nearly equivalent to the same fiber volume content of 30 wt.% glass fibers (see Table 3). To achieve a similar fiber volume content of cellulosic fibers, a 20 wt.% content was selected for this study. In order to investigate a comparison within one fiber type, compounds with 30 wt.% CRF and 40 wt.% GF were additionally prepared. The fiber weight content *ψ* and the fiber volume content *φ* were calculated mathematically according to the following formulas [51].

(1) $$\psi \left[wt.\%\right]=\frac{m_{Fiber}}{m_{Composite}}\times 100=\frac{m_{Fiber}}{m_{Fiber}+m_{Matrix}}\times 100$$

(2) $$\varphi \left[vol.\%\right]=\frac{\psi \times \rho_{Matrix}}{\psi \times \rho_{Matrix}-\left(1-\psi \right)\times \rho_{Fiber}}\times 100$$

In order to be able to evaluate in particular the influences of the extrusion process on the emission properties of the composites, the polyamide (without further additives and fibers) was extruded once. The emission properties were also determined from the polyamide (bag, dry) and the fibers (delivery state).

For the determination of the emission properties, the granules and fibers were examined before and the composites after extrusion. For the determination of the mechanical properties, standard test specimens were made from the produced composites in the injection molding process.

#### 2.2.1. Drying Processes before Processing and Humidity Control

Polyamides belong to the group of hygroscopic polymers, which absorb moisture and bind it physically within. Therefore, the residual moisture of the materials was determined before each processing step and the material was dried if necessary. The MA 100 Q moisture analyzer (Sartorius AG, Göttingen, Germany) was used to check the residual moisture.

The polyamide granule was delivered in moisture-proof 25 kg bags and could be processed without a further drying process. Drying of the granules was necessary only if the packaging had been damaged or the pack has been open for more than two hours. In this case, the material was dried in a Toro-Systems TR-Dry Jet air dryer (GfK Krämer GdbR, Igensdorf, Germany) at 80 °C for 1–4 h depending on the moisture content until the residual moisture content dropped below 0.1% again. Prior to the injection molding process, the composites with the glass fiber and cellulose fiber were dried by using the same process and parameters as the polyamide granule.

Due to the hydrophilic nature of the polymer, the cellulosic fiber also had to be dried, as the moisture content of the delivered fiber could be as high as 13%. Therefore, prior to the compounding process, the cellulosic fibers were dried in an air convection oven UT 20 P (Heraeus Holding, Hanau, Germany) at 105 °C until their moisture content was below 0.5%. The additional drying of the glass fiber was not necessarily due to the delivery condition (moisture content < 0.3%).

#### 2.2.2. Storage and Packaging of the Composites and Test Specimens

After compounding and injection molding, the materials and test specimens were stored batchwise in moisture-proof aluminum composite foil bags (ACF-bag). According to the respective standard, the samples were removed from the ACF-bag, conditioned, and tested in a standard climate (23 °C, 50% relative humidity). Instructions for conditioning are given in the respective test description.

#### 2.2.3. Compounding

**Figure 1:**
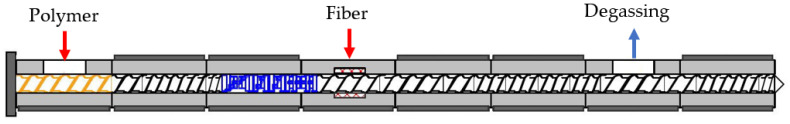
Used screw configuration of twin screw extruder.

The composites were carried out on a Leistritz ZSE 18 (Leistritz Extrusionstechnik GmbH, Nürnberg, Germany), a co-rotating twin screw extruder with a screw diameter of 18 mm and a process length of 40 D. The Leistritz ZSE 18 is used with a gentle screw configuration. The screw configuration simply consists of the conveying elements after the fiber feeding zone. The screw design is shown in Figure 1. The screw configuration was optimized in collaboration with Leistritz and used in processing in previous studies [14,33,52] for cellulose-based composites. The granules (feeding zone) and fibers (Zone 3) were fed by a gravimetric feeding system. A circular nozzle 3 mm in diameter was used for extrusion. The extruded strand was transported via an air-cooled conveyer belt and then granulated in a cutting mill Scheer SGS 25-E (Reduction Engineering GmbH, Stuttgart, Germany). The screw speed was set to 200 rpm and the material throughput was set to 2 kg/h. The screw configuration includes kneading discs and mixing elements to mix and distribute the fibers after they have passed through the feeding zone. The process temperatures of the compounding processes for the two different polyamides (PA 6.10 and PA 10.10) are shown in Table 4.

#### 2.2.4. Injection Molding

The test specimens (type 1A) were manufactured according to DIN EN ISO 527-1A [53] using an injection molding machine Arburg Allrounder 320C (Arburg GmbH & Co. KG, Loßburg, Germany) with a standard three-section screw with a diameter of 25 mm, and a clamping force of 50 kN. The injection speed was set to 16 cm^3^/s. The mold temperature was set to 80 °C and the inner mold pressure at injection was 800 bar. The cycle time was approx. 40 s, including a cooling time of 20 s. The processing temperatures for the two different polyamides (PA 6.10 and PA 10.10) are shown in Table 5. The temperature of the mold was set to 80 °C.

### 2.3. Characterization Techniques

#### 2.3.1. Tensile Tests According to DIN EN ISO 527

The tensile tests were carried out according to DIN EN ISO 527 [53] using the universal testing machine Zwick Z010 (Zwick Roell GmbH & Co. KG, Ulm, Germany). The specimen’s type 1A was tested with the testing speed of 5 mm/min. During the tests, the modulus, the tensile strength, and the elongation at break were evaluated. The test specimens were conditioned at 23 °C and a humidity of 50% for at least 24 h. At least seven specimens were tested for each composite.

#### 2.3.2. Instrumented Impact Strength Test According to DIN EN ISO 179-2

The instrumented Charpy impact tests were carried out according to DIN EN ISO 179-2 [54]. The test specimens were made from existing tensile test specimens and were sized to 10 mm × 4 mm × 80 mm with a 2 mm deep notch. The notches were inserted with a device by CEAST GmbH (Martinsried, Germany). The impact was performed on the narrow long side (e: edgewise impact) on the notched specimen (notch type A) from the direction of the unnotched side. An instrumented 5 J pendulum hammer was used to record force deformation curves. In instrumented impact testing, the pendulum hammer is equipped with electronic measuring instruments that can continuously record the force applied to the specimen as a function of time and the deformation of the specimen before fracture. This allows strain and stress to be recorded over the duration of the impact test. The data from the acquisition system provides a complete representation of the progression of the impact test rather than just a value from a single calculated number. The test samples were conditioned at 23 °C and a humidity of 50% for at least 24 h. Ten specimens were tested for each composite.

#### 2.3.3. Water Determination According to DIN EN ISO 15512

The Karl Fischer (KF) titration according to DIN EN ISO 15512 is used to determine the water content of the samples [55]. KF titration can be used to determine free and bound water, e.g., water absorbed in crystals on the surface or trapped inside. The Coulometer KF 756 (Deutsche Metronom GmbH & Co. KG, Filderstadt, Germany) was used to determine the water content. The oven temperature was 160 °C. Depending on the expected moisture content, the sample weighed between 50–100 mg. The tensile specimens were packed directly after the injection molding process and were tested in a freshly molded state. At least 2 specimens were tested for each composite. In case of large deviations, further samples were measured.

#### 2.3.4. GC-MS Analysis

The method of Thermal Desorption—Gas Chromatography—Mass Spectrometry (TD-GC-MS) was used in the analysis of qualitative as well as quantitative emissions in this study. The used GC-MS System 7890A GC System, 5975C inert XL MSD (Agilent Technologies, Santa Clara, CA, USA), the thermal desorption unit (TDU), and equipment for sampling (MPS) (Gerstel GmbH & Co.KG, Mühlheim an der Ruhr, Germany) form the unit of analysis. In order to determine emission from polyamide composites the regularly used method for interior material in the automobile industry is the VDA 278 method [56]. For the conditioning of the test specimens, the screening method for the determination of the emissions of volatile organic compounds from vehicle interior parts and materials—bag method (DIN ISO 12219-2) [57]—was carried out. Deviating from the standard, a temperature of 100 °C was selected for 7 days. These parameters were determined in preliminary tests. After conditioning, the test gas was transferred to a contained sorbent tube (sorbent: Tenax TA) by means of a vacuum pump. The samples (tenax tubes) are placed in a thermal desorption unit (TD) and VOCs and SVOCs are determined by thermal extraction. The samples (granule after extrusion) were subsequently analyzed with direct thermal desorption at 90 °C to determine VOC emissions. At least 2 specimens were tested for each composite. Analysis conditions are shown in Table 6.

The qualitative evaluation of the chromatograms is carried out using the “ChemStation” software from Agilent Technologies. The software is used to evaluate the peak areas and identify substances. For this purpose, the reference database of the spectra library of the National Institute of Standard and Technology (NIST) NIST 11 is used.

For quantitative evaluation, calibration runs are first carried out with the calibration solution (*toluene*). Subsequently, the VOC emission values can be calculated. Using the following formula, the response factor (*R_f_*) can first be calculated. The response factor results from the quotient of the absolute mass (in μg) of toluene and the resulting peak areas [52]:

(3) $$R_{f,VOC}=\frac{\mu g\;Toluene\;(C16)}{Peak\;area}\times 10^{6}$$

The quantitative *VOC* emission is calculated from the peak area of the emissions and the response factor of the reference substance calibration:

(4) $${Emission}_{VOC}\;[\mu g/g]=R_{f}\left(Toluene,C16\right)\times \frac{Peak\;area\;[Counts]}{sample\;test\;portion\;\left[mg\right]\times 10^{3}}$$

#### 2.3.5. Sensory Evaluation According VDA 270

The odor evaluation of the specimens was carried out in accordance with the VDA 270 standard [58]. This test method is used by various automotive suppliers and manufacturers to assess whether the materials can be used in the vehicle for interior applications. Contrary to the standard, granulate was used as a sample. The storage condition variant 3 was applied. According to the standard, 10 g (±1) of each sample was inserted in a 1 L glass vessel and kept closed at 80 °C for 2 h. After cooling to 60 °C, eight test persons evaluated the odor samples. The evaluation scale is shown in Table 7.

The arithmetic mean values of the ratings were calculated and being down rounded grades according to the VDA standard. Generally, the acceptance limit for materials intended for indoor applications is a test result up to 3.0 (for some car manufacturers the limit is 2.5).

#### 2.3.6. Sensory Evaluation According to EN 13725

Dynamic olfactometry, as described by the European standard EN 13725, has become the preferred method for evaluating odor emissions [59]. The olfactometer TO8 (Olfasense GmbH, Kiel Germany) was used. The samples (granules) were inserted in bags made of ^®^Nalophan (polyethylene terephthalate). A total of 40 g (±1) of each sample was inserted in the sample bag, filled with four liters of nitrogen, and kept closed at 80 °C for 2 h (according to VDA 270). The panel comprised 4–6 assessors which were qualified according to DIN EN 13725. At least four measurements per panel were carried out for each composite. The odor concentration was calculated according to DIN EN 13725. Prior to the analysis, all data were converted by logarithmic (base-10) transformation to the odor level with the unit decibel (db) analogous to the noise level which is also given in the unit db.

Considering that the research involved human subjects, the university’s central ethics committee reviewed the project and assessed safety-relevant research risks. The investigations were classified as unproblematic in terms of research ethics, so the study may be submitted to the funding institution and its results can be published.

## 3. Results and Discussion

### 3.1. Mechanical Properties

**Figure 2:**
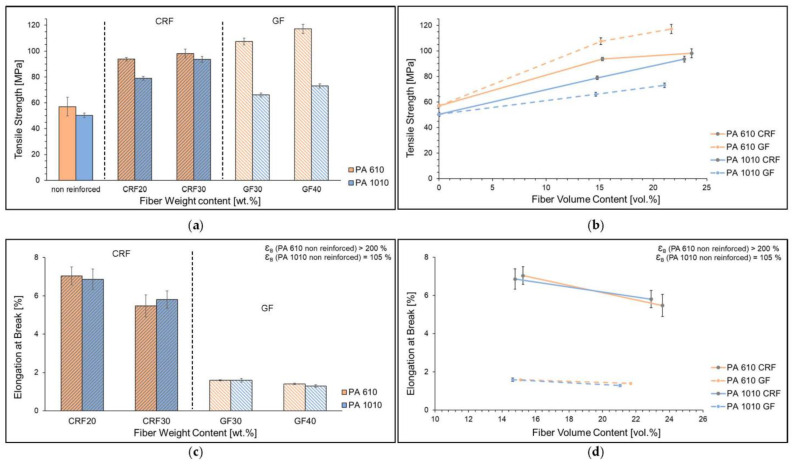
Tensile strength (**a**,**b**) and Elongation at Break (**c**,**d**) of reinforced composites (CRF and GF) with different fiber content of PA 6.10 and PA 10.10 matrices depending on the fiber weight content (**a**,**c**) and the fiber volume content (**b**,**d**).

The tensile strength and elongation at the break of reinforced PA 6.10 and PA 10.10 are shown in Figure 2. In this investigation, tensile strengths of up to 94 MPa (20 wt.% CRF) and 98 MPa (30 wt.% CRF) were achieved for regenerated cellulose-reinforced PA 6.10. Using PA 10.10, tensile strengths could be achieved up to 79 MPa (20 wt.% CRF) and 93 MPa (30 wt.% CRF) and were, thus, lower than the PA 6.10 composites. The results show an increase in tensile strength with the increase in fiber content. Erdmann [32] concluded from its investigations that, in general, a clear reinforcing effect can be observed due to the fiber component. However, the degree of the effect varies depending on the polyamide type, the polarity, and the CH_2_/CO-NH ratio. While with reinforcement using PA 6.10, the tensile strengths with the glass fibers reached values of 107 to 117 MPa and are, therefore, higher than the cellulosic fiber related to the comparable fiber volume content. The decrease in tensile strength of the PA 10.10 composites is striking. Contrary to the investigations by Feldmann [23] and Gemmeke [33], the tensile strengths of the glass fiber composites are lower than those of the cellulose fiber composites with the same fiber weight or fiber volume content. This cannot be clarified here but is probably due to the manufacturing conditions in the injection molding process. The reinforcement with cellulosic and glass fibers results in contrast, that the elongation decreases with increasing fiber reinforcement. The elongation at break of the composites of the various used matrices does not differ significantly. The elongation at break of PA 6.10 (above 200%) and PA 10.10 (105%) decreases to below 7% (CRF) and to be low 2% for the glass fiber-filled composites.

**Figure 3:**
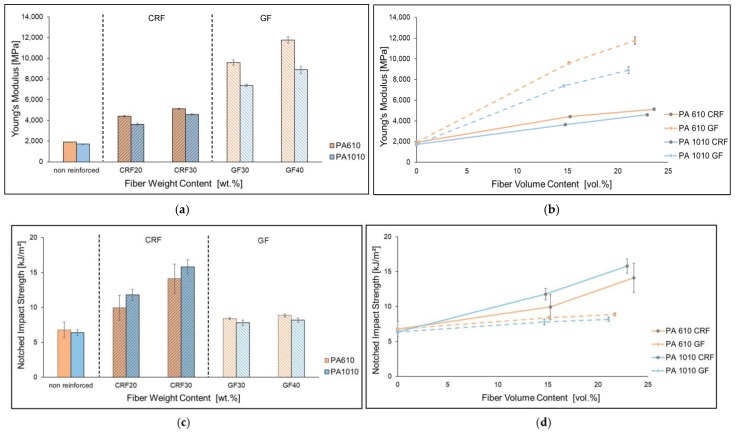
Young’s Modulus (**a**,**b**) and notched impact strength (**c**,**d**) of reinforced composites (CRF and GF) with different fiber content of PA 6.10 and PA 10.10 matrices depending on the fiber weight content (**a**,**c**) and the fiber volume content (**b**,**d**).

Figure 3 shows Young’s Modulus and the charpy notched impact strength of PA 6.10 and PA 10.10 and the fiber-reinforced composites. Young’s Modulus increases with an increased fiber content of cellulosic fiber. A further increase in Young’s Modulus can be observed due to the fiber reinforcement with the glass fiber. Overall, the Young’s Modulus of the composites with PA 10.10 as the matrix material is between approx. 10–25% lower than that of the PA 6.10 composites. Impact strength increases with increasing cellulose fiber content, while glass fiber reinforcement does not show much difference with increasing fiber content. The investigations of von Ganster and Gemmeke also found that the impact characteristics increase drastically using regenerated cellulose fiber as reinforcement. Glass fiber reinforced composites have a lower notched impact strength compared to regenerated cellulose fiber reinforced composites [27,33]. PA 10.10 with the cellulose fiber reinforcement achieves up to 16% (CRF20) higher values than with the PA 6.10 matrix. 

**Figure 4:**
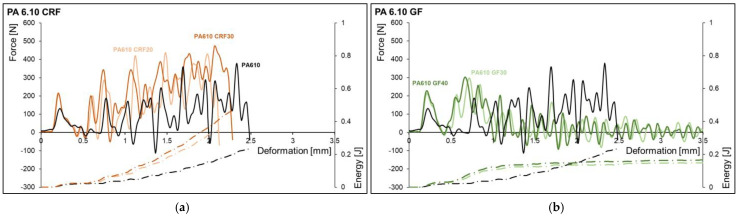
Force-deformation curves and energy-deformation curves of the notched charpy impact tests of the matrix material PA 6. 10 and the composites with cellulosic fiber (**a**) and glass fiber (**b**).

**Figure 5:**
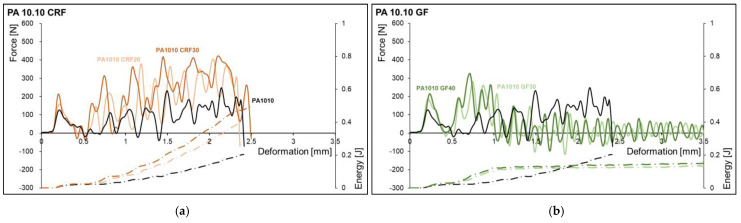
Force-deformation curves and energy-deformation curves of the notched charpy impact tests of the matrix material PA 10.10 and the composites with cellulosic fiber (**a**) and glass fiber (**b**).

The force-deformation curves and the energy deformation curve of PA 6.10 and PA 10.10 with different fibers and fiber contents are shown in Figure 4 and Figure 5. The samples of the matrix materials (PA 6.10 and PA 10.10) and the cellulose fiber reinforced composite to show a complete break. For the glass fiber-reinforced composites, partial breaks and hinge breaks were observed as the failure types. An increase in deformation until breakage could only be observed with PA 10.10 CRF30. The deformation to break (partial, hinge) is lower for glass fiber-reinforced materials than for cellulose fiber reinforced composites. The highest forces were studied for cellulosic fiber-reinforced composites. The significantly higher elongation at the break of the cellulose fiber leads to higher deformation and higher energy absorption. It has not been observed that the higher strength and modulus of the glass fibers result in higher forces in the glass fiber-reinforced composites compared to the cellulose fibers.

### 3.2. Moisture—Water Content of Specimens

**Figure 6:**
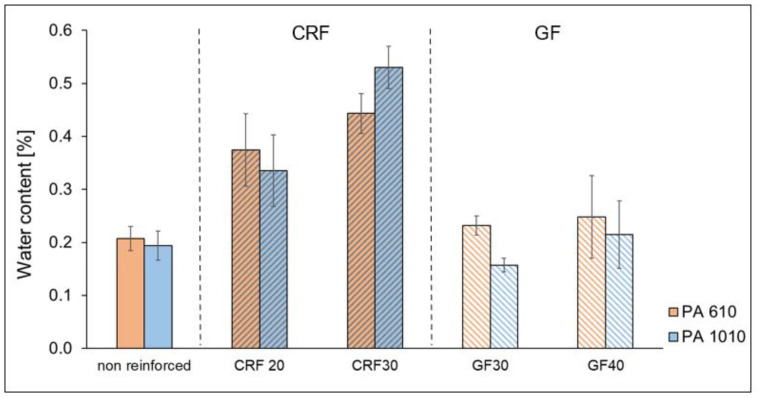
Water content of PA 6.10 and PA 10.10 materials and composites (tensile test specimens).

The water content of PA 6.10 and PA 10.10 and the composites with cellulosic and glass fiber are shown in Figure 6. For the composites with cellulose fiber, the water content increases with increasing fiber content and is significantly higher than the water content of the matrix material. The water content of the glass fiber-reinforced composites is around the level of the matrix material. With the exception of the composites with a cellulose fiber content of 30 wt.%, the water content of the PA 10.10 composites is lower than that of the PA 6.10 variants. Cellulose fibers are generally known for their rapid moisture absorption. Feldmann observed that a dry cellulose fiber (moisture < 0.2 wt.%) absorbs up to 3 wt.% moisture within one hour and up to 8 wt.% moisture after 24 h in a standard climate (23 °C/50%) [23]. However, the fibers were dried, and the attention was paid not to expose the fibers to ambient air for a long time, it can be assumed that moisture absorption during handling (extrusion, injection molding process, and storage of the granules and specimens until cooling and packaging) cannot be completely prevented.

### 3.3. VOC Emission of Materials and Composites

#### 3.3.1. Quantitative Evaluation of the VOC Emission Values

Preliminary tests have shown that the sample preparation method using the bag method according to DIN ISO 12219-2 and the analytical method according to VDA 278 are suitable for the investigation. Furthermore, the evaluation of the compounds was performed using the calibration standard (toluene) for VOC emissions. Before the calculation procedure, a couple of values were noted during the preparation of calibration standard toluene. The calibration was performed in double determination. In the evaluation software, the ion chromatograms of the calibration runs were integrated, analyzed, and interpreted. The results are shown in Table 8.

Below are the results of the emission tests for materials 1–14. Chemical substances are compared to a series of tests conducted. Table 9 shows the results of the material and composite emissions test with their corresponding emission values.

**Figure 7:**
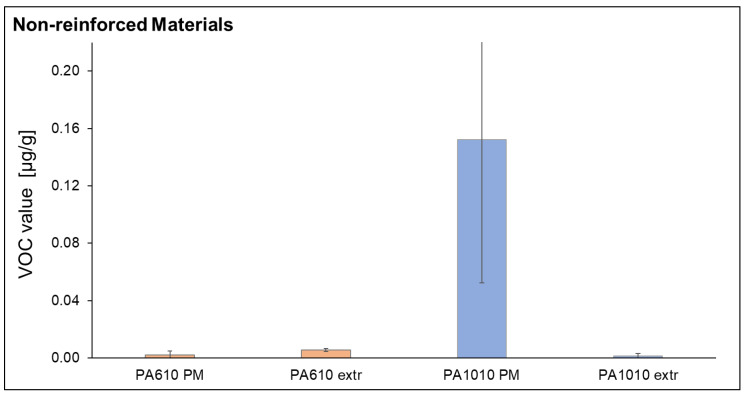
VOC Emission values of non-reinforced materials (PA PM and PA extr) of both used matrices.

The comparison of the VOC emissions of polymer materials without fibers is shown in Figure 7. The polymer material PA 10.10 which was taken directly from the airtight packaging and the cellulosic fiber has the highest value of VOC emissions of all examined composites and materials before the extrusion process. For PA 10.10, several measurements from different bags were repeated due to the conspicuously high values. The high values could be confirmed but cannot be explained here. For the polymer material PA 6.10, emissions increase slightly after extrusion.

**Figure 8:**
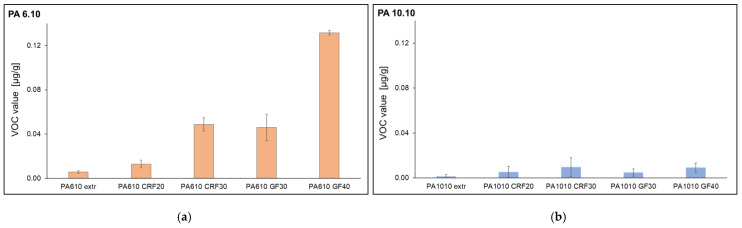
VOC Emission values of PA 6.10 (**a**) and PA 10.10 (**b**) composites.

In the following, the VOC emissions of the composites are compared with the VOC emissions of the matrix materials (one-time extruded) in order to consider the influence of the fiber addition. Figure 8 shows the VOC emissions of PA 6.10 and PA 10.10, the fiber-reinforced composites with cellulosic fiber and glass fiber. The VOC emissions increase with increasing fiber content. The VOC emissions of glass-fiber-filled PA 6.10 are higher than those of cellulose composites which can be compensated by the fiber volume content. For the extruded matrix materials, PA 10.10 shows lower VOC emission than PA 6.10. For the PA 10.10 composites, a slight increase in VOC emissions with increasing fiber content can also be observed. These results may indicate that the addition of fiber content to both materials (PA 10.10 and PA 6.10) has an apparent effect on the emission value. According to Rhodes et al., parameters that can influence emissions are melt temperature, extrusion rate, shear effects due to screw design, and others [60]. This would explain the higher emissions for the PA 6.10 composites because they were processed at higher temperatures. Feldmann found that significant temperature-induced bulk degradation of a regenerated cellulose fiber was observed between 240–260 °C. However, the first damage begins at temperatures above 175 °C [23]. Moll has also found that temperature and moisture content have a significant influence on emissions. In addition, the residual monomer content of a substance is the cause of VOC emissions [61]. Additionally, Moll noted that due to thermal and thermal-oxidative stresses during extrusion, the material undergoes chemical reactions that can produce volatile degradation products that contribute to increased emissions. Accordingly, temperature changes affect both the quantity and spectrum of compounds released: the higher the temperature, the faster the emission process, and the higher boiling substances are released [61]. This statement fits perfectly with the analyzed substances since the materials during extrusion are processed at different temperatures.

#### 3.3.2. Qualitative Evaluation of the VOC Emissions

The identification of the detected substances was assigned by comparing the retention times (R_t_) of the chromatographic peak from the emission samples. The peak area (Pa) is specified in percent and refers to the proportion of all detected substances in the measurement of a sample. Only those substances were listed that could be determined with a probability of at least 80%. In the table, CAS No. (Chemical Abstract Service Registry Number), the substance type, and typical odor of the substances were also listed. The information was obtained from the NIST database (National Institute of Standards and Technology, U.S Department of Commerce, Gaithersburg, MD, USA) [62], the PubChem database (National Center for Biotechnology Information, Bethesda, MD, USA) [63], and the GESTRIS-Material database [64].

##### Glass Fiber and Regenerated Cellulose Fiber

**Figure 9:**
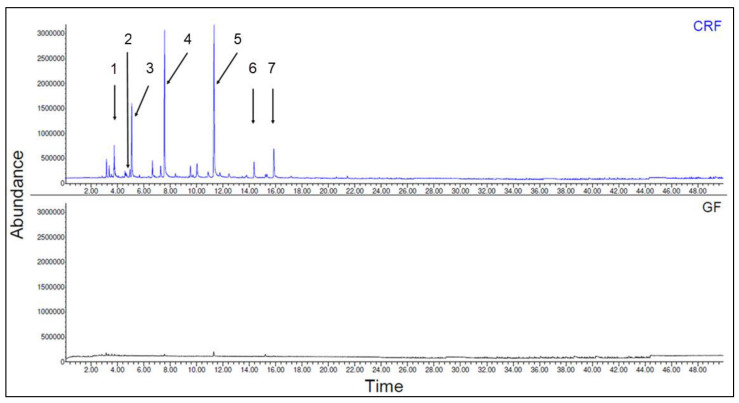
Emission spectra of cellulose fibers (CRF) and glass fibers (GF) (the designation of substances 1–7 and further information are listed in the following table).

The emission spectra of regenerated cellulosic fiber (CRF) and glass fiber (GF) are shown in Figure 9. Glass fibers show no signs of emissions in the chromatogram (no peaks), which indicates an emissions value of 0 µg/g. However, cellulose fibers show a tremendous amount of peak formation in the first 16 minutes of retention. The detected and identified substances of cellulosic fiber are listed in Table 10.

##### Polymer Matrix PA 6.10 and PA 6.10 Composites

**Figure 10:**
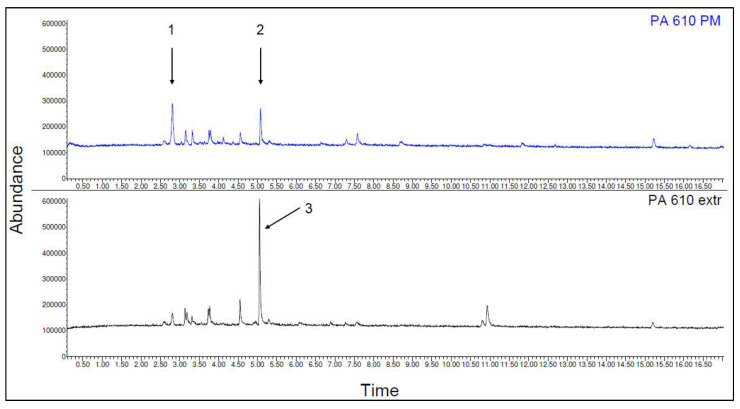
Emission spectra of the polymer PA 610 (packaged material (PM) and one time extruded (extr) (the designation of substances 1–3 and further information are listed in the following table).

The emission spectra of PA 6.10 packaged material (PM) and one time extruded (extr) are shown in Figure 10. The detected and identified substances of PA 6.10 are listed in Table 11.

**Figure 11:**
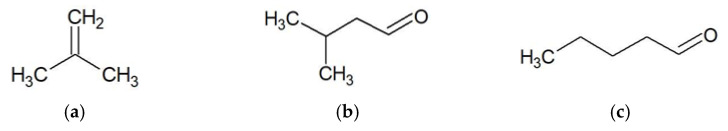
Chemical structures of substances found in PA 6.10 [63]: (**a**) Chemical structure of 1-propene, 2-methyl; (**b**) Chemical structure of butanal, 3-methyl; (**c**) Chemical structure of pentanal.

Before the processing of the material, the two substances 1-propene, 2-methyl- (C_4_H_8_), and butanal, 3-methyl- (C_5_H_10_O) could be detected. After one-time extrusion, only the substance pentanal (C_5_H_10_O) could be detected. The chemical structures of the substances are shown in Figure 11.

**Figure 12:**
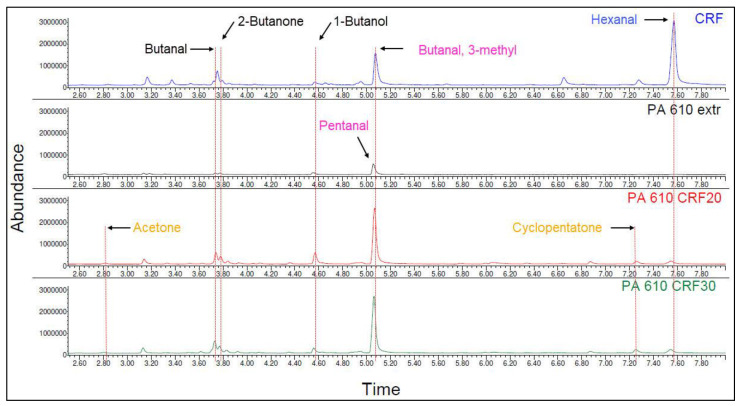
Emission spectra of PA 6.10 composites with regenerated cellulose fibers (CRF).

The emission spectra of the regenerated cellulose fiber, PA 6.10 one-time extruded (extr), and the composites with the cellulose fiber are shown in Figure 12. The relevant detected and identified substances are listed in Table 12.

Acetone and cyclopentanone can only be detected in the composites. Butanal, 2-butanol, and 1-butanol are detected in the fiber and the composites. As will be shown in the following, these substances also occur in the composites with glass fibers, which leads to the assumption that the substances are formed by the stress on the melt due to the presence of fibers and do not originate from the fibers themselves. Pentanal and butanal, 3-methyl have a group of four isomeric saturated aldehydes with five carbon atoms. They have the general molecular formula C_5_H_10_O and a molar mass of 86.13 g/mol. The chain length is identical, but the arrangement of the atoms is different as shown in Figure 6. As VOCs with the same chain length, they are close to each other but differ significantly in their properties (e.g., melting point and boiling point) [61]. Hexanal is detected in fiber and composites, therefore it can be assumed that processing does not completely degrade hexanal like the other substances detected in the fiber (see Table 9).

**Figure 13:**
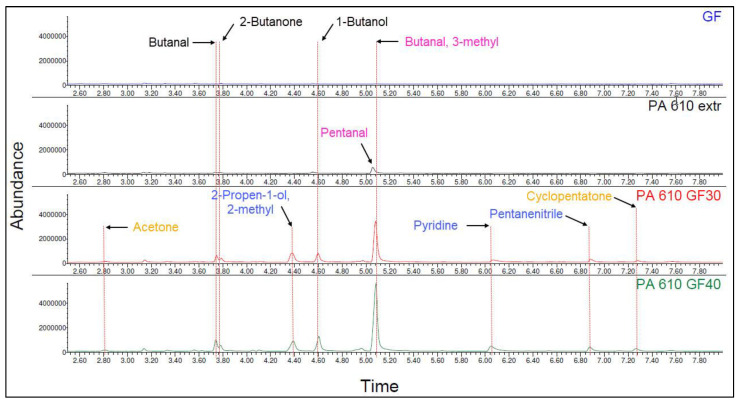
Emission spectra of PA 6.10 Composites with glass fibers (GF).

The emission spectra of the glass fiber, PA 6.10 one time extruded (extr), and the composites with the glass fiber are shown in Figure 13. The detected and identified substances are listed in Table 13.

No substances could be detected in the glass fiber. 2-Propen-1-ol, 2-methyl-, pyridrine, and pentane nitrile are detected only in the composites reinforced with glass fiber. 2-Propen-1-ol, 2-methyl-, and pentane nitrile are used in the chemical industry. More precise information on the use of glass fibers in the production process could not be found. Pyridine bases are used in glass fiber production as a lubricant (auxiliary agent in fiber production) which protects the fiber against impact and pressure as well as electrostatic charging before further processing [62,63]. In contrast to the cellulose fiber composites, the hydrocarbon compounds C_5_H_10_O are found in the form of butanal, 3-methyl- in the glass fiber composites.

##### Polymer Matrix PA 10.10 and PA 10.10 Composites

**Figure 14:**
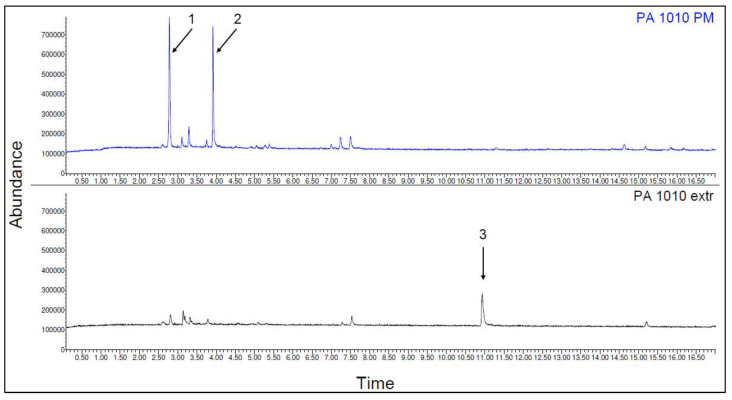
Emission spectra of polymer PA 10.10 bag material (PM) and one-time extruded (extr) (the designation of substances 1–3 and further information are listed in the following table).

The emission spectra of PA 10.10 packaged material (PM) and one-time extruded (extr) are shown in Figure 14. The identified VOCs of PA 10.10 are listed in Table 14.

**Figure 15:**
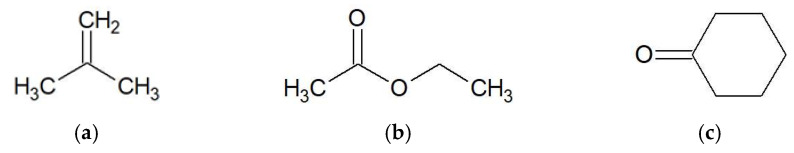
Chemical structures of substances found in PA 6.10 [63]: (**a**) Chemical structure of 1-propene, 2-methyl; (**b**) Chemical structure of ethyl acetate; (**c**) Chemical structure of cyclohexanone.

Before the processing of the material, the two substances 1-propene, 2-methyl- (C_4_H_8_), and ethyl acetate (C_4_H_8_O) could be detected. After one-time extrusion, only the substance Cyclohexanone (C_5_H_8_O) could be detected. The chemical structures of the substances are shown in Figure 15.

In PA 10.10 composites, only a few substances could be detected. None of the detected substances of the composites could be detected in advance in the fibers or PA 10.10. The relevant detected and identified substances are listed in Table 15 and Table 16.

In summary, it can be stated that in contrast to glass fiber reinforced polypropylene (HC: C6-C31) [47], only hydrocarbon compounds (HC) with a chain length of up to C6 have been detected.

It is crucial to consider moisture absorption when dealing with polyamides, as it affects the mechanical properties and the geometrical dimensions to a large extent. A material’s strength declines during elongation, and toughness increases with high moisture content. Therefore, moisture content affects many properties. CH_2_/CONH says something about how much moisture a polyamide usually absorbs. The greater the distance between polar groups, the less water is absorbed, and the greater its strength, stiffness, and impact resistance, and the greater its impact resistance. Polyamides with an even number of C-atoms (i.e., PA 6, PA 6.10, etc.) have higher intermolecular forces because the CO- and NH-groups are positioned opposite. In addition, their melting points are higher as a result. A polyamide with an uneven number of C-atoms, on the other hand, has a relatively low melting point, but a higher impact strength [32,35].

As already mentioned, moisture has an impact on the movement of molecules, which in turn affects the spectrum of substances [61]. The two types of polyamides used are different regarding the length of the diamine or dicarboxylic acid. Based on the chemical structure of both types, it has been proven that the ratio of non-polar CH_2_ groups to the polar amide groups (CO-NH) influences moisture content. The smaller the ratio, the more polar the PA type is, which also means higher intermolecular interactions. Therefore, an increasing polarity means higher melting temperatures and higher water absorption capacity [32]. Table 17 shows the ratio value of CH_2_/CO-NH groups and their moisture content.

Based on the facts mentioned above regarding temperature and moisture and their effect on emissions, it is safe to say that they confirm the analysis fluctuations between PA 6.10 and PA 10.10. PA 6.10 has a higher emissions value than PA 10.10, which may be intensely relatable to the mentioned causes.

### 3.4. Odor Evaluation

In a supplementary study, the odor properties of selected composites were investigated as a function of extruder speed (n_1_ = 150 rpm, n_2_ = 300 rpm) and different temperature profiles (T_1_ see Table 4 and T_2_ see Table 18). Extreme processing conditions were to be generated with the speed n_2_ and the temperature profile T_2_ in order to investigate whether this significantly changes the odor properties.

**Figure 16:**
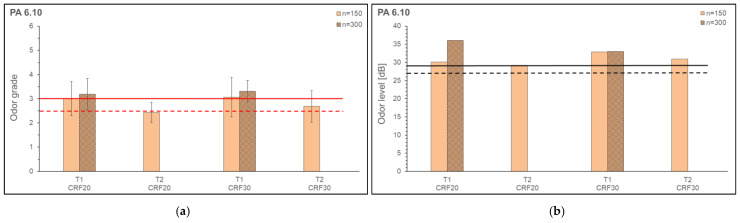
Odor Grade (**a**) and Odor Level (**b**) of cellulose reinforced PA 6.10 with different fiber contents.

**Figure 17:**
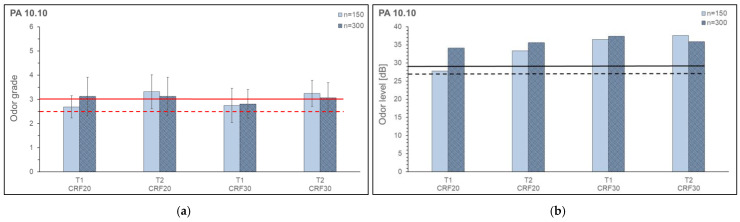
Odor Grade (**a**) and Odor Level (**b**) of cellulose reinforced PA 10.10 with different fiber contents.

The odor grade and odor level of reinforced PA 6.10 and PA 10.10 are shown in Figure 16 and Figure 17. For the odor investigations, the speed (n_1_, n_2_) and two temperature profiles (T_1_, T_2_) were varied. Generally, the acceptance limit for materials intended for indoor applications is an odor grade of up to 3.0 (for some car manufacturers the limit is 2.5). These two limit values are marked in the figures (odor grade). For an evaluation of the odor level, limit values from Maiwald were used [65]. Considering a maximum expanded measurement uncertainty, maximum measured values of 500 GE/m^3^ (27 db) for clean gas and 800 GE/m^3^ (29 db) for biofilters are given, for example. These two limit values are marked in the figures (odor level). The samples for cellulose-reinforced PA 6.10 at n_2_ and T_2_ could not be produced (composite showed burnt spots on extruder strand) due to the high melt temperature in combination with the cellulosic fiber and are, therefore, not included in the evaluation.

The odor grades and also the odor level of the cellulose-reinforced composites are in the range of the limit values or above. The odor grades of the composites with PA 6.10 and PA 10.10 do not differ significantly. The odor levels of PA 10.10 composites are on average higher than the odor levels of PA 6.10 composites. The determined VOC emissions (Section 3.3) of the composites are in a range of 0.0013–0.13150 µg/g on a relatively low level, nevertheless, they are clearly perceived by the panel during the odor evaluation. This may be due to the fact that the emitted substances have a high odor intensity and contribute greatly to the overall odor of the sample.

## 4. Conclusions

The investigation evaluated the potential of regenerated cellulose fibers as a reinforcement in bio polyamide (PA 6.10, PA 10.10) and show the results in comparison to common glass fiber reinforced composites. The influence of the fiber and the fiber content on VOC and odor emission (granules) and the mechanical properties, of injection molded specimens were studied.

- Compounding bio-based PA and regenerated cellulose fibers (Cordenka) with a conventional twin-screw extruder and gravimetric feeding system was realized with 20 wt.% and 30 wt.% fiber content;
- Compounding bio-based PA with glass fibers (30 wt.% and 40 wt.%) was used as a comparable volume share for comparisons;
- With the combination of the modified bag method (DIN ISO 12219-2) and TD-GC-MS according to VDA 278, substances can be successfully identified, and VOC emissions quantified;
- VOC emissions (quantitative) were at a low level compared to other studies (other materials and composites);
- The results of the odor tests showed values mostly above the required limit values;
- The Young’s Modulus and the Impact strength increase with increasing fiber content. PA 6.10 and PA 10.10 achieve significantly higher impact strengths with the regenerated cellulose fibers than the composites with glass fibers;
- Compared to glass fiber reinforced PA 6.10 and PA 10.10, a significantly higher elongation at break with regenerated cellulose fibers can be achieved;
- While a further increase in tensile strength was observed for PA 6.10 with the use of glass fibers, the glass fiber-reinforced PA 10.10 composites had significantly lower tensile strengths in comparison with regenerated cellulose fibers;
- The highest forces were studied for cellulosic fiber-reinforced composites. The significantly higher elongation at the break of the cellulose fiber leads to higher deformation and higher energy absorption.
- The investigations showed that the composites investigated can be used in automotive interiors due to their mechanical properties. Due to the high elongation at the break of the cellulose fiber, a significantly higher impact strength of the PA 6.10 and PA 10.10 composites was achieved. In the passenger compartment, cellulose fiber-reinforced bio polyamides can, thus, be used in particular for crash-relevant components. In view of the results of the odor tests, the values are not yet sufficient for use in automobile interiors.
- Further investigations to optimize the processing parameters in extrusion (optimization of the screw configuration, an adaptation of the process parameters, etc.) and in the injection molding process (residence time, process temperature, etc.) could provide solutions.

The method for determining the emissions could be adapted for comparative measurements and to optimize the process parameters. An increase in the thermo-desorption-temperature for the TD-GC-MS method is conceivable; this would be a deviation from VDA 278, but could possibly replace the lengthy bag method and lead to shorter analysis times overall. Moreover, a method, such as, e.g., dynamic headspace (DHS), would be conceivable to make time-optimized measurements possible.

## Figures and Tables

**Table 1:** Details and characteristics of Vestamid Terra DS 16 and HS 16 used for the production of fiber reinforced bio-polyamide composites [9,23].

Properties	PA 10.10 (DS16) Dry/Cond.	PA 6.10 (HS16)	Unit	Test Standard	Source
Share of renewable material	100	up to 62	wt.%	-	[9]
Tensile Modulus	1700/1250	2100	MPa	ISO 527-1/-2	[9]
Yield stress	54/48	61	MPa	ISO 527-1/-2	[9]
Yield strain	2/22		%	ISO 527-1/-2	[9]
Density	1.04/-	1.08	g/cm^3^	ISO 1183	[9]
Melting temperature	200	222	°C	ISO 11357-1/-3	[9]
Glass transmission temperature	54	60	°C	DMA	[9]
Melt Flow Rate (230 °C/5 kg)	90	-	g/10 min	DIN EN ISO 1133	[23]
Melt Flow Rate (250 °C/5 kg)	130	131	g/10 min	DIN EN ISO 1133	[23]
Monomer content	0.74	0.84	%	by extraction	[23]
Relative viscosity	1.64–1.77	1.64–1.77	-	DIN EN ISO 307	[23]

**Table 2:** Characteristics of the used glass fiber (GF) and the regenerated cellulose fiber (CRF) [23,48,49,50].

Properties	GF	CRF	Unit
Fiber length	3	2	mm
Fiber diameter	10	12–15	µm
Density	2.5	1.5	g/cm^3^
Modulus	73	20	GPa
Strength	3400	830	MPa
Elongation at break	3.5–4	13	%
Decomposition temperature	-	from 175	°C
Softening temperature	840	-	°C
Moisture content	max. 0.3	* approx. 13	%
Smell	odorless	odorless	-

* (20 °C/65% rel. hum.).

**Table 3:** Investigated materials and composites.

Material and Composites	Fiber Type	Polymer Weight Content [wt.%]	Fiber Weight Content [wt.%]	Fiber Volume Content [vol.%]
CRF	Cellulose	0	100	100
GF	Glass	0	100	100
PA 610 PM (packaged material)	-	100	0	0
PA 610 extr (one time extruded)	-	100	0	0
PA 610 CRF20	Cellulose	80	20	15.2
PA 610 CRF30	Cellulose	70	30	23.5
PA 610 GF30	Glass	70	30	15.1
PA 610 GF40	Glass	60	40	21.1
PA 1010 PM (packaged material)	-	100	0	0
PA 1010 extr (one time extruded)	-	100	0	0
PA 1010 CRF20	Cellulose	80	20	14.7
PA 1010 CRF30	Cellulose	70	30	22.9
PA 1010 GF30	Glass	70	30	14.6
PA 1010 GF40	Glass	60	40	21.7

**Table 4:** Processing temperatures in the compounding process (PA 610 an PA 1010).

Temp. [°C]	Feeding Zone	Zone 1	Zone 2	Zone 3	Zone 4	Zone 5	Zone 6	Zone 7	Nozzle
PA 610	50	240	240	230	230	230	220	210	220
PA 1010	50	220	220	200	180	180	180	180	205
Function	Feeding polymer	Melting	Melting	Feeding fiber	Mixing	Homo- genisation	Vacuum Degassing	Pressure Build-up	Sharping

**Table 5:** Processing temperatures in injection molding process for (PA 6.10 an PA 10.10).

Temp. [°C]	Feeding Zone	Zone 1	Zone 2	Zone 3	Zone 4	Zone 5
PA 6.10	50	200	215	225	225	245
PA 10.10	50	190	205	215	225	225

**Table 6:** Analysis parameter of GC-MS method according VDA 278.

Parameters	Values
TDU (Thermal Desorption Unit)	splitless, desorption flow 82.3 mL/min
	20 °C (0.5 min); 60 °C/min;
	90 °C (30 min)
	280 °C transfer heater
CIS (Cooled Injection System)	split 100:1
	−150 °C (0.5 min); 12 °C/s; 280 °C (5 min)
GC Column	HP-5 ms UI; 5% Phenyl Methyl Si
	l = 30 m; di = 250 μm; df = 0.5 μm
Pneumatics	He, constant flow = 3 mL/min
Oven	40 °C (2 min); 10 °C/min; 280 °C (5 min)
MSD (Mass Spectrometry Device)	Scan, 29–450 amu

**Table 7:** Evaluation scale according to VDA 270.

Grade 1	Grade 2	Grade 3	Grade 4	Grade 5	Grade 6
not perceptible	perceptible, not disturbing	clearly perceptible, but not disturbing	disturbing	strongly disturbing	not acceptable

**Table 8:** Result of VOC calibration—Calculated response factor (Rf).

Run	Ret. Time [min]	Peak Area [counts]	Start Time [min]	End Time [min]
1	5.201	125,356,435	5.190	5.401
2	5.201	137,707,589	5.190	5.390
	Mean peak area	$\overset{-}{x}$ = 131,532,012		
	Response factor	*R_f,VOC_* = 0.015327		

**Table 9:** Result of VOC analysis—Mean Value VOC Emission of two runs.

Sample No.	Material and Composites	VOC Emission [µg/g]
1	PA 610 PM	0.00200
2	PA 610 extr	0.00575
3	PA 610 CRF20	0.01294
4	PA 610 CRF30	0.04875
5	PA 610 GF30	0.04598
6	PA 610 GF40	0.13150
7	PA 1010 PM	0.15245
8	PA 1010 extr	0.00130
9	PA 1010 CRF20	0.00535
10	PA 1010 CRF30	0.00980
11	PA 1010 GF30	0.00495
12	PA 1010 GF40	0.00925
13	Glass Fiber (GF)	0.00000
14	Regenerated Cellulose Fiber (CRF)	0.16440

**Table 10:** GC peak library of regenerated cellulose fibers.

No.	Substance	R_t_ [min]	CAS No.	Characteristic (Type; Odor; Use; VOC)
1	Butanal	3.756	123-72-8	aldehyde; characteristic, pungent, aldehydic odor; VOC
*	2-Butanone	3.796	078-93-3	ketone, moderately sharp, mint- or acetone-like odor; VOC
*	1-Butanol	4.566	071-36-3	alcohol, harsh fusel odor with banana, rancid, sweet; VOC
2	1-Heptene	4.952	592-76-7	alkene; petroleum-like odor; VOC
3	Pentanal	5.079	110-62-3	aldehyde; strong, acrid, pungent odor, smells of bready, fruity, nutty, berry; VOC
4	Hexanal	7.572	66-25-1	aldehyde; smell of fresh lawn clippings or apple mash; VOC
5	1-Heptanal	11.324	111-71-7	aldehyde; penetrating fruity to oily greasy odor; VOC
6	1-Heptanol	14.354	111-70-6	alcohol, faint aromatic alcohol odor; VOC
7	Octanal	15.875	124-13-0	aldehyde; starting material for fragrance, rose oil, lemon oil; VOC

* The substances do not reach the 80% probability (only 58%), but they do play a role in the composite analysis. Therefore, they are listed in this table.

**Table 11:** GC peak library of the polymer PA 610 (packaged material (PM) and one time extruded (extr).

No.	Substance	R_t_ [min]	CAS No.	Characteristic (Type; Odor; Use; VOC)
1	1-Propene, 2-methyl-	2.811	115-11-7	alkene, faint petroleum-like odor; VOC
2	Butanal, 3-methyl-	5.084	590-86-3	aldehyde; apple-like odor and a powerful penetrating, acrid odor; VOC
3	Pentanal	5.060	110-62-3	aldehyde; strong, acrid, pungent odor, smells of bready, fruity, nutty, berry; VOC

**Table 12:** GC peak library of the PA 6.10 composites with regenerated cellulose fibers (CRF).

Substance	CAS No.	PA 610 PM PA 610 Extr		CRF		PA610 CRF 20		PA610 CRF30		Hydrocarbon Compounds		Other Atoms
		R_t_ [min]	Pa [%]	R_t_ [min]	Pa [%]	R_t_ [min]	Pa [%]	R_t_ [min]	Pa [%]	C- Atoms	H- Atoms	
1-Propene, 2-methyl-	115-11-7	2.811	59.8							C4	H8	
Acetone	067-64-1					3.142	4.34	3.132	4.79	C3	H6	O
Butanal	123-72-8			3.756	3.73	3.742	8.16	3.732	10.50	C4	H8	O
2-Butanone	078-93-3			* 3.796	* 0.85	3.786	4.91	3.771	4.49	C4	H8	O
1-Butanol	071-36-3			* 4.566	* 0.81	4.571	10.88	4.562	3.73	C4	H10	O
Pentanal	110-62-3	5.060	100.0							C5	H10	O
Butanal, 3-methyl-	590-86-3	5.084	40.2	5.079	10.62	5.069	55.87	5.064	61.08	C5	H10	O
Cyclopentanone	120-92-3					7.260	3.12	7.255	4.60	C5	H8	O
Hexanal	066-25-1			7.572	25.00	7.543	4.37	7.543	5.49	C6	H12	O

* The substances do not reach the 80% probability (only 58%). (Substances detected in PA 610 Extr).

**Table 13:** GC peak library of the PA 6.10 composites with glass fibers (GF).

Substance	CAS No.	PA 610 PM PA 610 Extr		GF		PA610 GF 30		PA610 GF 40		Hydrocarbon Compounds		Other Atoms
		R_t_ [min]	Pa [%]	R_t_ [min]	Pa [%]	R_t_ [min]	Pa [%]	R_t_ [min]	Pa [%]	C-Atoms	H-Atoms	
1-Propene, 2-methyl-	115-11-7	2.811	59.8							C4	H8	
Acetone	067-64-1					3.147	4.79	3.142	1.28	C3	H6	O
Butanal	123-72-8					3.747	4.92	3.742	5.96	C4	H8	O
2-Butanone	078-93-3					3.786	3.04	3.786	2.91	C4	H8	O
2-Propen-1-ol, 2-methyl-	513-42-8					4.381	13.47	4.391	10.26	C4	H8	O
1-Butanol	071-36-3					4.601	9.60	4.605	10.63	C4	H10	O
Pentanal	110-62-3	5.060	100.0							C5	H10	O
Butanal, 3-methyl-	590-86-3	5.084	40.2			5.084	45.16	5.084	53.37	C5	H10	O
Pyridine	110-86-1					6.069	5.04	6.050	6.84	C5	H5	N
Pentanenitrile	110-59-8					6.889	3.58	6.889	3.52	C5	H9	N
Cyclopentanone	120-92-3					7.280	2.29	7.265	2.26	C5	H8	O

(Substances detected in PA 610 Extr).

**Table 14:** GC peak library of the polymer PA 10.10 bag material (PM) and one time extruded (extr).

No.	Substance	Retention Time [min]	CAS No.	Characteristic (Type; Odor; Use; VOC)
1	1-Propene, 2-methyl-	2.785	115-11-7	alkene, faint petroleum-like odor; VOC
2	Ethyl acetate	3.917	141-78-6	ester; fruity smell of pineapple; VOC
3	Cyclohexanone	10.934	108-94-1	ketone, peppermint or acetone-like odor; production of nylon, as a chemical reaction medium; VOC

**Table 15:** GC peak library of the PA 10.10 composites with regenerated cellulose fibers (CRF).

Substance	CAS No.	PA 1010 PM PA 1010 Extr		CRF		PA1010 CRF 20		PA1010 CRF30		Hydrocarbon Compounds		Other Atoms
		R_t_ [min]	Pa [%]	R_t_ [min]	Pa [%]	R_t_ [min]	Pa [%]	R_t_ [min]	Pa [%]	C- Atoms	H- Atoms	
1-Propene, 2-methyl-	115-11-7	2.785	58.4							C4	H8	
Acetone	067-64-1							3.132	4.79	C3	H6	O
Butanal	123-72-8			3.756	3.73					C4	H8	
Ethyl acetate	141-78-6	3.917	41.96							C4	H8	O
Pentanal	110-62-3			5.079	10.62					C5	H10	O
Octane	111-65-9					7.538	100.0	7.543	71.92	C8	H18	
Hexanal	066-25-1			7.572	25.00					C6	H12	O
Cyclohexanone	108-94-1	10.94	100.0							C6	H10	O

**Table 16:** GC peak library of the PA 10.10 composites with glass fibers (CRF).

Substance	CAS No.	PA 1010 PM PA 1010 Extr		GF		PA1010 GF 30		PA1010 GF 40		Hydrocarbon Compounds		Other Atoms
		Rt [min]	Pa [%]	Rt [min]	Pa [%]	Rt [min]	Pa [%]	Rt [min]	Pa [%]	C- Atoms	H- Atoms	
1-Propene, 2-methyl-	115-11-7	2.785	58.4							C4	H8	
2-Propanol, 2-methyl	075-65-0					3.342	21.00	3.342	12.63	C4	H10	O
2-Butanone	078-93-3							3.801	9.75	C4	H8	O
Ethyl acetate	141-78-6	3.917	41.96							C4	H8	O
2-Propen-1-ol, 2-methyl-	513-42-8					4.367	36.29			C4	H8	O
Octane	111-65-9					7.558	42.1	7.543	71.92	C8	H18	
Cyclohexanone	108-94-1	10.94	100.0							C6	H10	O

(Table 15 and Table 16: Substances detected in PA 1010 Extr).

**Table 17:** Different types of PA with their respective CH_2_/CO-NH ratio and moisture content (according to [23]).

PA-Type	Ratio CH_2_/CO-NH	Moisture [%]
PA 6.10	7	3.3
PA 10.10	9	1.8

**Table 18:** Processing temperatures T_2_ in the compounding process (PA 610 and PA 1010).

Temp. [°C]	Feeding Zone	Zone 1	Zone 2	Zone 3	Zone 4	Zone 5	Zone 6	Zone 7	Nozzle
PA 610	50	240	240	230	230	230	220	220	230
PA 1010	50	240	240	230	230	230	220	210	220
Function	Feeding polymer	Melting	Melting	Feeding fiber	Mixing	Homogenisation	Vacuum Degassing	Pressure Build-up	Sharping

## Data Availability

Not applicable.
